# Association of Apolipoprotein E (APOE) Polymorphisms With Serological Lipid and Inflammatory Markers

**DOI:** 10.7759/cureus.60721

**Published:** 2024-05-20

**Authors:** Hari K. Krishnamurthy, Imbaasree Rajavelu, Swarnkumar Reddy, Michelle Pereira, Vasanth Jayaraman, Karthik Krishna, Qi Song, Tianhao Wang, Kang Bei, John J Rajasekaran

**Affiliations:** 1 Biomedical Engineering, Vibrant Sciences LLC, San Carlos, USA; 2 Clinical Research, Vibrant America LLC, San Carlos, USA; 3 Research & Development, Vibrant Sciences LLC, San Carlos, USA; 4 Data Acquisition and Analysis, Vibrant America LLC, San Carlos, USA; 5 Data Acquisition and Analysis, Vibrant Sciences LLC, San Carlos, USA

**Keywords:** e4/e4, e3/e3, apoe genotypes, apolipoprotein e, alzheimer's disease, cardiovascular risk, hscrp, ldl, inflammation, lipids

## Abstract

Background

The study aims to assess the association of apolipoprotein E (APOE) gene polymorphisms with serological lipid and inflammatory markers to determine their potential role in predicting the risk of cardiovascular diseases (CVDs) and Alzheimer's disease (AD).

Methodology

A total of 915 individuals underwent testing for lipid and inflammatory biomarkers at Vibrant America Clinical Laboratory. Clinical data, blood lipid and inflammatory profiles, and APOE genotyping were analyzed using polymerase chain reaction-restriction fragment length polymorphism (PCR-RFLP).

Results

Compared to the E3/E3 genotype, individuals with E2/E3 genotypes showed higher levels of high-density lipoprotein (HDL), triglycerides, apolipoprotein A (APOA), high-sensitivity C-reactive protein (hs-CRP), and myeloperoxidase (MPO). E2/E4 genotype carriers had higher levels of HDL, triglycerides, Lp(a), and N-terminal pro b-type natriuretic peptide (BNPNT). E3/E4 genotypes were associated with elevated levels of total cholesterol, LDL, Lp(a), hs-CRP, small-density low-density lipoprotein (SDLDL), oxidized LDL (OXLDL), MPO, LDL-CAL, PLAC, and APOB. The E4/E4 group displayed higher concentrations of total cholesterol, LDL, APOB, Lp(a), hs-CRP, SDLDL, OXLDL, MPO, LDLCAL, and PLAC compared to E3/E3 carriers. These findings highlight the potential atherogenic effect of the ε4 allele and the protective effect of the ε2 allele based on lipid and inflammatory marker profiles.

Conclusions

This study provides strong evidence linking APOE gene polymorphism to abnormal serum lipid and inflammatory profiles. Individuals carrying the ε4 alleles exhibited dysregulated lipid metabolism and abnormal inflammatory markers, increasing their risk of CVD and AD. Early detection and prompt diagnosis are crucial for implementing therapeutic, dietary, and lifestyle interventions to mitigate risks and prevent or delay lipid and inflammation-related disorders.

## Introduction

Apolipoprotein E (APOE) is a lipoprotein encoded by the APOE gene located on chromosome 19q13.32 and is composed of 299 amino acids and weighs 34 kDa [1]. The APOE gene has three common alleles at the APOE locus, namely, ε2, ε3, and ε4, which give rise to six major genotypes: three homozygous (APOE2/2, APOE3/3, and APOE4/4) and three heterozygous (APOE2/3, APOE2/4, and APOE3/4). These genotypes differ in the amino acids at positions 112 and 158, resulting in the production of APOE2 (Cys112; Cys158), APOE3 (Cys112; Arg158), and APOE4 (Arg112; Arg158). APOE3/3 is the most common genotype occurring in approximately 60%-75% of individuals, followed by APOE2/3 and APOE3/4, which account for about 15%-25% and 10%-20% of the population, respectively [2]. The APOE4/4 genotype, associated with a higher risk of disease, is less prevalent, occurring in approximately 2%-15% of individuals, depending on the population studied [3]. The variations in APOE genotypes lead to differences in APOE isoforms, which, in turn, influence various cellular functions in the central nervous system (CNS) and peripheral tissues [4,5]. 

In the CNS, APOE isoforms have distinct effects on different cell types. Astrocytes are the primary source of APOE synthesis, although microglia and neurons synthesize this protein in certain circumstances [6]. The APOE produced by different cell types in the CNS is subsequently lipidated by the ATP-binding cassette transporter A1 (ABCA1) to form lipoprotein particles [7,8]. APOE isoforms exhibit differential abilities of binding/transporting cholesterol and phospholipids, with APOE2 having the highest ability, followed by APOE3, and APOE4 (APOE2 > APOE3 > APOE4). Consequently, APOE4 is poorly lipidated compared with APOE2 and APOE3 [9]. APOE4 specifically disrupts lipid metabolism and transport ability in astrocytes, potentially leading to neuronal dysfunction and neurodegeneration. Microglial cells expressing APOE4 show an amplified pro-inflammatory response compared to those expressing APOE3, indicating a role for APOE isoforms in modulating immune responses in the CNS [10,11]. Understanding the effects of APOE isoforms on different cell types in the CNS is crucial for studying neurological disorders. 

In peripheral tissues, APOE is predominantly produced in the liver, with minor contributions from the adrenal gland and macrophages [12]. Peripheral APOE is then incorporated into lipoprotein particles in the plasma. In plasma, APOE is specifically associated with very low-density lipoprotein (VLDL), chylomicron remnants, and a subset of high-density lipoprotein (HDL) particles [13]. It is a high-affinity ligand for the low-density lipoprotein (LDL) receptor as well as its family members including the LDL receptor-related protein (LRP1), VLDL receptor, and APOE2 receptor (LPR8) as it interacts with these receptors to promote the endocytic clearance of plasma lipoproteins, especially VLDL and remnant lipoproteins [13]. Peripheral APOE plays a critical role in regulating lipid metabolism and has profound implications for cardiovascular disease (CVD) function and systemic inflammation. Notably, APOE isoforms influence the binding of APOE to different lipoprotein particles, such as VLDLs, LDLs, and HDLs, leading to variations in their metabolism [14]. APOE4 exhibits a preference for binding to VLDLs and LDLs, while APOE3 shows a higher affinity towards HDLs [15]. Furthermore, genetic variation in the APOE gene has been found to affect inflammation by influencing serum C-reactive protein (CRP) levels [16]. The intricate interactions between APOE isoforms, lipoproteins, receptors, and inflammatory signaling pathways contribute to the complex regulation of lipid metabolism and inflammation in peripheral tissues. These interrelated processes have implications for the development and progression of CVD and the overall systemic inflammatory status [17]. 

Previous studies have reported that lipid markers such as LDL, HDL, small-density LDL (SDLDL), and oxidized LDL (OXLDL) are affected by APOE polymorphism. APOE4 has been linked to higher levels of LDL and SDLDL particles, while APOE2 has been associated with lower LDL and higher SDLDL levels [18,19]. Additionally, APOE4 is believed to correlate with lower levels of HDL, and APOA markers while APOE2 is associated with high levels of the same. APOE4 has also been associated with increased susceptibility to OXLDL-induced oxidative stress and inflammation [18,19]. Further, inflammatory markers such as high-sensitivity CRP (hs-CRP) and myeloperoxidase (MPO) are influenced by the APOE polymorphism. APOE4 has been linked to higher levels of hs-CRP associated with CVD, as well as MPO, which is associated with oxidative stress and inflammation [20].

With evidence suggesting that the APOE polymorphisms play a significant role in the modulation of serological lipid and inflammatory markers, effective analysis must be carried out to elucidate the mechanism underlying these effects. The study aims to provide a comprehensive analysis of the APOE polymorphism and its effects on lipid and inflammatory markers. Understanding the interplay of the APOE polymorphisms with lipid metabolism and inflammation will pave the way for early risk assessment and the implementation of personalized interventions for mitigating the risk of CVD and AD. 

## Materials and methods

Study population 

The study population comprised 916 individuals who were tested for vital lipid and inflammatory biomarkers at Vibrant America Clinical Laboratory. The study was exempted from formal ethical reviews by Western IRB (Work order #1-1098539-1) (Washington, USA) since the study comprises the retrospective analysis of deidentified clinical data and test results. 

APOE genotyping 

For genotyping, genomic DNA was isolated from blood samples, and genetic analysis was carried out by the PCR-RFLP method using a real-time PCR system. 

Determination of serum lipids and inflammatory markers 

Blood samples were collected and processed to obtain serum. Total cholesterol levels were measured using an enzymatic assay catalyzed by cholesterol dehydrogenase and estimated on the Beckman Coulter AU680 analyzer. Serum levels of apolipoprotein A (APOA), apolipoprotein B (APOB), high-sensitivity C-reactive protein (hs-CRP), and lipoprotein(a) (Lp(a)) were estimated using a particle-enhanced immunoturbidometric assay on the Roche Cobas 6000 C 501 analyzer. LDL, HDL, SDLDL, and triglyceride levels in serum were measured using an enzyme-based colorimetric assay on the Beckman Coulter AU680 analyzer. N-terminal pro-b-type natriuretic peptide (BNPNT) levels were determined through an electrochemiluminescence immune assay. Total serum homocysteine (HOMOC) levels were estimated using an enzyme-linked immunosorbent assay on the Beckman Counter AU series Analyzers. OXLDL levels were quantified using a sandwich technique that uses two monoclonal antibodies specific to antigenic determinants on the oxidized APOB. The reaction was based on the reaction between the OXLDL in the serum with anti-oxidized LDL antibodies, the reaction is monitored spectrometrically at 450 nm. Serum myeloperoxidase (MPO) was determined by a latex-enhanced immuno-turbidimetric assay based on antigen-antibody interaction. The MPO in the serum binds to a specific anti-MPO antibody coated on the latex. This causes agglutination and the turbidity of agglutination is directly measured as the concentration of MPO in the serum. The in vitro quantification of the lipoprotein-associated phospholipase (Lp-PLA2) test (PLAC) is an enzymatic assay based on the hydrolysis of the sn-2 position of the substrate and produces a colored product 4-nitrophenol. The change in the absorbance with the production of 4-nitrophenol is measured as the Lp-PLAC activity. The concentration of LDL calculated (LDLCAL) was computed using triglycerides and HDL concentration using Friedewald’s formula (LDLCAL = total cholesterol − HDL cholesterol − (triglycerides/5)). 

Statistical analysis 

Statistical analysis was performed using Microsoft Excel (version 2021). Descriptive statistics were used to summarize the characteristics of the study population, including mean and standard deviation for continuous variables. To examine the association between the APOE genotypes and the lipid and inflammatory profile, t-tests were performed. The means of the marker levels were compared among genotypes. Pearson correlation coefficients were calculated to examine the relationship between each lipid and inflammatory profile variable and the APOE genotypes. The level of statistical significance was set at *P* < 0.05. The results of the statistical analysis were reported as mean ± standard deviation and correlation coefficients as appropriate. 

## Results

The current research aimed to study the association of APOE genotypes with circulating levels of vital lipid markers including total cholesterol, LDL, HDL, triglycerides, APOA, APOB, Lp(a), BNPNT, SDLDL, OXLDL, APOB:APOA, and LDLCAL, and inflammatory markers, including hs-CRP, HOMOC, MPO, and PLAC. 

Baseline characteristics of lipid and inflammatory markers 

This study evaluated the association of 12 lipids and four inflammatory markers. The overall study population included 594 (64.84%) female and 322 (35.2%) male subjects, with the mean age of females being 50.89 ± 15.83 years, while that for males being 50.90 ± 15.83 years. The frequency of the six genotypes analyzed in the study was E3/E3 (*N* = 594, 64.84%), E2/E2 (*N *= 1, 0.001%), E2/E3 (N = 94, 10.26%), E2/E4 (*N *= 20, 2.183%), E3/E4 (*N *= 188, 20.52%), and E4/E4 (*N *= 19, 2.07%). The baseline characteristics of lipid and inflammatory markers of the subjects studied are given in Table 1. Among the lipid markers, elevated levels were observed for cholesterol (193.06 ± 41.25 mg/dL), while lower levels were found for APOB:APOA (0.60 ± 0.18 U/L). In terms of the inflammatory markers, higher levels were detected for MPO (1143.84 ± 995.12 pmol/L), whereas lower levels were observed for hs-CRP (2.79 ± 6.38 mg/dL). 

**Table 1:** Baseline characteristics of lipid and inflammatory markers. Gender distribution is depicted as a percentage, with males and females, respectively. APOE genotype frequency is displayed as a percentage, showing the proportion of each genotype (ε2/ε2, ε2/ε3, ε2/ε4, ε3/ε3, ε3/ε4, and ε4/ε4) within the study population. Marker levels are expressed as mean ± standard deviation (SD). LDL, low-density lipoprotein; HDL, high-density lipoprotein; APOA, apolipoprotein A; APOB, apolipoprotein B; Lp(a), lipoprotein A; hs-CRP, high-sensitivity C-reactive protein; HOMOC, homocysteine; BNPNT, B-type natriuretic peptide; SDLDL, small-density LDL; OXLDL, oxidized low-density lipoprotein;  MPO, myeloperoxidase; LDLCAL, LDL calculated; PLAC, lipoprotein-associated phospholipase (Lp-PLA2) test

Characteristics	
Gender in percentage	
Female (*N *= 594)	64.84%
Male (*N *= 322)	35.2%
Age (Mean ± SD) (years)	
Female (*N *= 594)	50.89 ± 15.83
Male (*N *= 322)	50.90 ± 15.83
APOE genotype frequency	
E3/E3 (*N *= 594)	64.84%
E2/E2 (*N *= 1)	0.001%
E2/E3 (*N *= 94)	10.26%
E2/E4 (*N *= 20)	2.183%
E3/E4 (*N *= 188)	20.52%
E4/E4 (*N *= 19)	2.074%
Parameters (mmol/L)	
Cholesterol (mg/dL)	193.06 ± 41.25
LDL (mg/dL)	124.44 ± 36.66
HDL (mg/dL)	60.35 ± 17.87
Triglycerides (mg/dL)	97.05 ± 47.60
APOA (mg/dL)	168.29 ± 35.19
APOB (mg/dL)	97.44 ± 24.03
Lp(a) (mg/dL)	37.63 ± 38.37
hs-CRP (mg/dL)	2.79 ± 6.38
HOMOC (µmol/L)	9.72 ± 3.17
BNPNT (pg/mL)	93.43 ± 226.50
SDLDL (mg/dL)	29.33 ± 11.08
OXLDL (U/L)	45.99 ± 20.38
MPO (pmol/L)	1143.84 ± 995.12
APOB:APOA (U/L)	0.60 ± 0.187
LDLCAL (mg/dL)	113.29 ± 35.09
PLAC (ng/mL)	164.08 ± 43.42

Effects of APOE polymorphism on the lipid and inflammatory profile 

**Figure 1:**
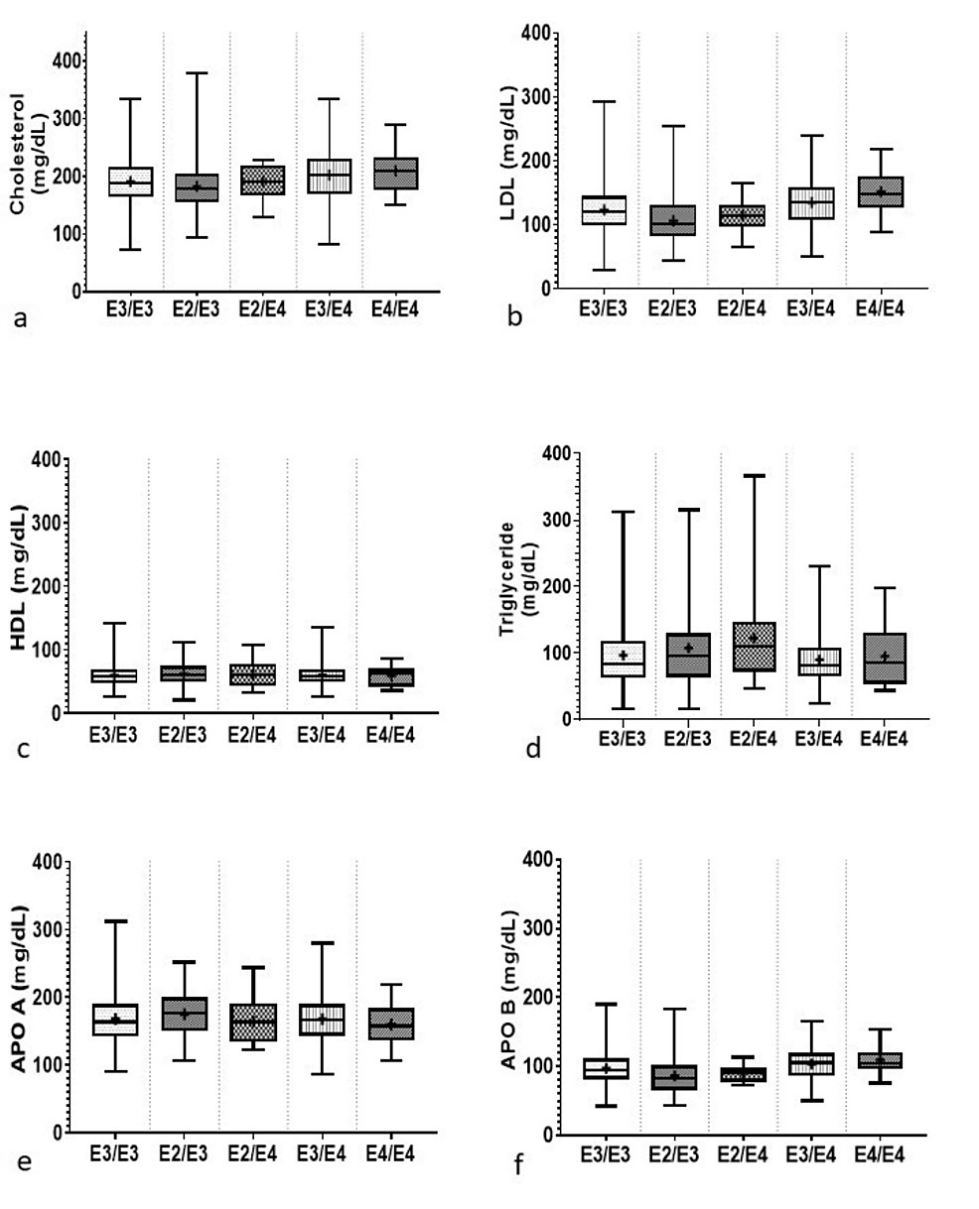
Distribution of serum biomarkers with respect to APOE genotype: (a) Lp(a); (b) hs-CRP; (c) homocysteine, (d) b-type natriuretic peptide; (e) SDLDL; (f) OXLDL. Box plots depicting the distribution of serum biomarkers across different APOE genotypes. Each box represents the interquartile range (IQR), with the median marked by a horizontal line within the box. Whiskers extend to 1.5 times the IQR from the first and third quartiles, with outliers plotted as individual points beyond the whiskers. Data are represented as mean ± standard deviation (SD). Statistical significance was determined using t-tests for comparison of means among genotypes and Pearson correlation coefficients for assessing the relationship between biomarkers and APOE genotypes. The level of statistical significance was set at *P* < 0.05. Lp(a), lipoprotein A; hs-CRP, high-sensitivity C-reactive protein; BNPNT, b-type natriuretic peptide; SDLDL, small-density LDL; OXLDL, oxidized low-density lipoprotein

**Figure 2:**
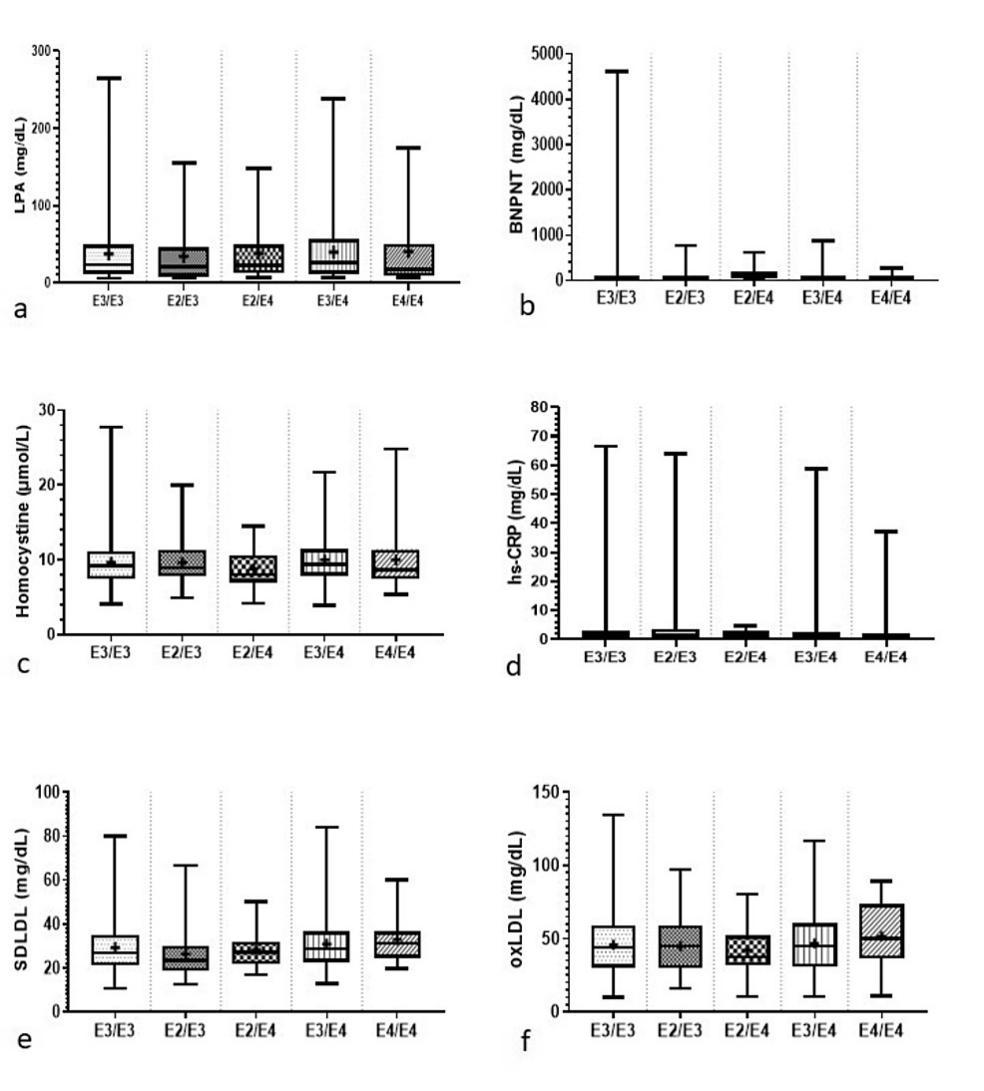
Distribution of serum biomarkers with respect to APOE genotype: (a) MPO; (b) APOB:APOA; (c) LDLCAL; (d) Lp-PLA2. Box plots illustrating the distribution of serum biomarkers across different APOE genotypes. Each box plot represents the distribution of biomarker levels among different genotypes of APOE. The horizontal line within each box represents the median value, while the box itself encompasses the interquartile range (IQR). Whiskers extend to the minimum and maximum values within 1.5 times the IQR. Outliers are shown as individual data points. Statistical significance was determined using t-tests, with significance levels set at *P* < 0.05, indicating significant differences between groups. Descriptive statistics are presented as mean ± standard deviation. Pearson correlation coefficients were calculated to examine the relationship between each biomarker and APOE genotypes. MPO, myeloperoxidase; LDLCAL, LDL calculated; PLAC, lipoprotein-associated phospholipase (Lp-PLA2) test

**Figure 3:**
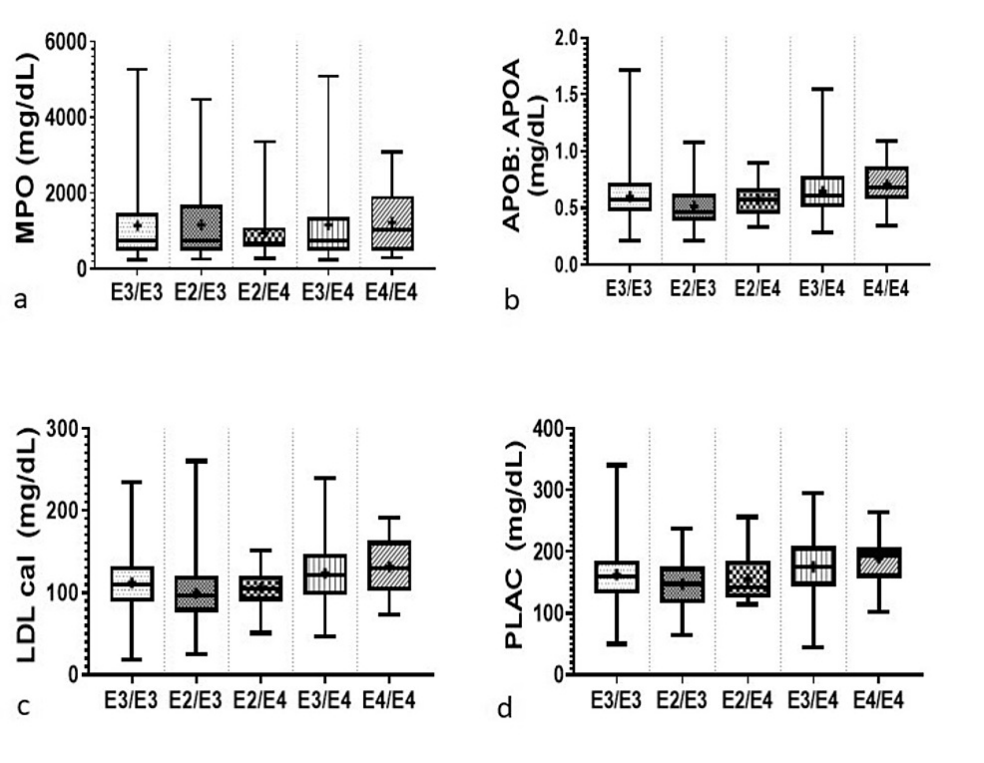
Distribution of serum biomarkers with respect to APOE genotype: (a) cholesterol; (b) LDL; (c) HDL; (d) triglycerides; (e) apolipoprotein A; (f) apolipoprotein B. Box plot illustrating the distribution of serum biomarkers. Each box plot depicts the median (line within the box), interquartile range (IQR, box edges), and range of the data (whiskers). The vertical axis represents the concentration levels of the respective biomarkers, while the horizontal axis denotes the different APOE genotypes. Statistical parameters are presented as mean ± standard deviation (SD). Statistical significance was determined using a significance threshold of *P* < 0.05. LDL, low-density lipoprotein; HDL, high-density lipoprotein; APOA, apolipoprotein A; APOB, apolipoprotein B

The mean values for the lipid and inflammatory parameters in association with the APOE genotypes for the study population are presented together in Table 2. The box plot represents the distribution of lipid and inflammatory markers studied with respect to APOE genotypes in Figure 1, Figure 2, and Figure 3, respectively. On comparing with the most commonly occurring genotype, E3/E3, it was found that individuals carrying the E2/E3 genotypes had higher levels of HDL, triglycerides, APOA, and hs-CRP, while individuals carrying the E2/E4 genotype had higher levels of HDL, triglycerides, Lp(a), and BNPNT. Individuals carrying the E3/E4 genotypes had higher levels of total cholesterol, LDL, Lp(a), hs-CRP, SDLDL, OXLDL, MPO, LDL-CAL, PLAC, and APOB when compared to those carrying the E3/E3 genotype. Individuals in the E4/E4 group had higher concentrations for total cholesterol, LDL, APOB, Lp(a), hs-CRP, SDLDL, OXLDL, MPO, LDLCAL, and PLAC compared with E3/E3 carriers. 

**Table 2:** Baseline characteristics of serological lipids and inflammatory markers by APOE genotypes. Data are represented as mean ± SD for continuous variables, including cholesterol, LDL, HDL, triglycerides, APOA, APOB, Lp(a), HSCRP, HOMOC, BNPNT, SDLDL, OXLDL, MPO, APOB:APOA, LDLCAL, and PLAC. Statistical significance was determined using t-tests for comparison of means. A *P*-value less than 0.05 was considered statistically significant. LDL, low-density lipoprotein; HDL, high-density lipoprotein; APOA, apolipoprotein A; APOB, apolipoprotein B; Lp(a), lipoprotein A; hs-CRP, high-sensitivity C-reactive protein; HOMOC, homocysteine; BNPNT, B-type natriuretic peptide; SDLDL, small-density LDL; OXLDL, oxidized low-density lipoprotein; MPO, myeloperoxidase; LDLCAL, LDL calculated; PLAC, lipoprotein-associated phospholipase (Lp-PLA2) test

	E3/E3	E2/E3	E2/E4	E3/E4	E4/E4
Parameters (mmol/L)	Mean ± SD	Mean ± SD	Mean ± SD	Mean ± SD	Mean ± SD
Cholesterol (mg/dL)	191.2 ± 40.64	182.95 ± 42.2	191.25 ±28.56	202.36 ± 42.487	209.89 ± 38.76
LDL (mg/dL)	123.5 ± 35.96	106.50 ± 36.7	114.55 ± 24.50	134.84 ± 35.60	151.78 ± 32.47
HDL (mg/dL)	59.8 ±17.47	62.18 ± 18.77	60.56 ± 21.028	60.75 ± 18.41	59.18 ± 14.32
Triglycerides (mg/dL)	96.72 ± 47.58	107.9 ± 58.28	122.7 ± 74.79	90.23 ± 36.03	95.32 ± 43.98
APOA (mg/dL)	167.7 ± 35.36	174.78 ± 34.56	164.09 ± 34.28	167.56 ± 34.89	159.64 ± 30.9
APOB (mg/dL)	97.2 ± 23.52	86.29 ± 25.35	90.51 ± 12.51	103.61 ± 23.69	109.26 ± 20.96
Lp(a) (mg/dL)	37.3 ± 38.66	34.16 ± 34.39	38.15 ± 39.806	39.74 ± 38.56	40.22 ± 47.74
hs-CRP (mg/dL)	2.65 ± 5.87	3.56 ± 7.58	1.71 ± 1.41	2.89 ± 7.38	3.27 ± 8.33
HOMOC (µmol/L)	9.67 ± 3.20	9.63 ±2.72	8.80 ± 2.57	10.00 ± 3.16	9.99 ± 4.77
BNPNT (pg/mL)	94.9 ± 267.15	85.39 ±99.76	135.9 ± 144.63	90.23 ± 130.41	75.77 ± 78.86
SDLDL (mg/dL)	29.2 ± 10.92	26.34 ± 11.19	28.36 ± 8.01	30.86 ± 11.56	32.88 ± 10.74
OXLDL (U/L)	45.8 ± 20.56	44.8 ± 17.67	42.36 ± 18.07	46.74 ± 20.99	51.58 ± 24.36
MPO (pmol/L)	1141.8 ± 1013.76	1156.6 ± 927.7	953.9 ± 754.05	1160.3 ± 1012.1	1215.8 ± 879.4
APOB:APOA (U/L)	0.60 ± 0.18	0.51 ± 0.18	0.57 ± 0.151	0.64 ± 0.19	0.70 ±0.18
LDLCAL (mg/dL)	112.01 ± 34.07	99.19 ± 36.9	106.11 ± 22.58	123.56 ± 35.306	131.67 ± 33.96
PLAC (ng/mL)	162.68 ± 42.28	147.26 ± 38.29	155.54 ± 40.44	175.66 ± 45.72	189.81 ± 44.46

Pearson correlation of APOE genotypes with vital lipid and inflammatory biomarkers

**Figure 4:**
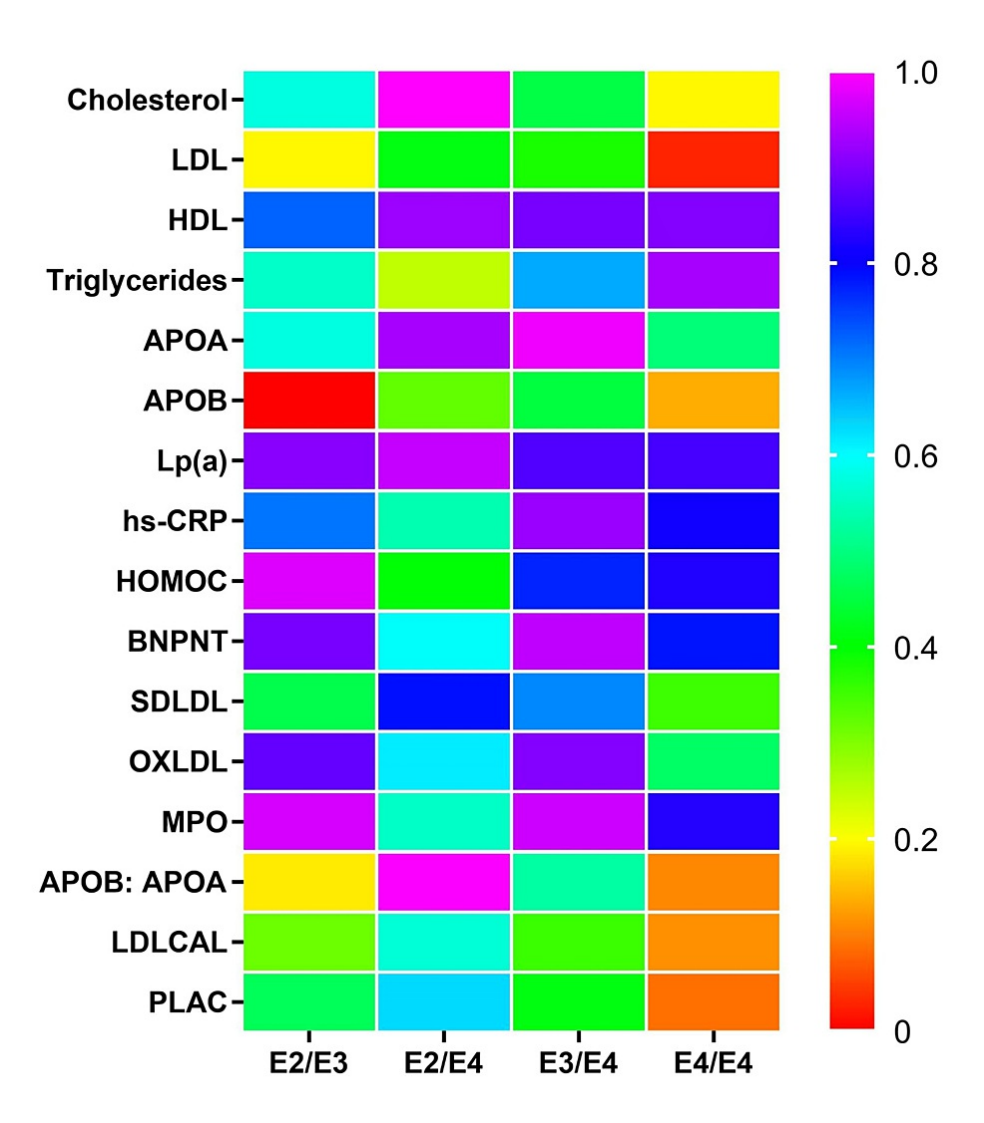
Heat map for Pearson correlation of serum lipids and inflammatory markers with APOE genotypes. The correlation coefficients are represented by a color gradient, with warmer colors indicating stronger positive correlations and cooler colors indicating stronger negative correlations. Statistical significance was determined at the level of *P* < 0.05. Data are represented as Pearson correlation coefficients. LDL, low-density lipoprotein; HDL, high-density lipoprotein; APOA, apolipoprotein A; APOB, apolipoprotein B; Lp(a), lipoprotein A; hs-CRP, high-sensitivity C-reactive protein; HOMOC, homocysteine; BNPNT, B-type natriuretic peptide; SDLDL, small-density LDL; OXLDL, oxidized low-density lipoprotein;  MPO, myeloperoxidase; LDLCAL, LDL calculated; PLAC, lipoprotein-associated phospholipase (Lp-PLA2) test

The current study used Pearson correlation coefficients to examine the associations between different genotypes and the E3/E3 genotype in terms of the biomarker profile. The results are presented in Table 3, and the heat map is plotted to visualize the correlations in Figure 4. Here, we discuss the correlation of the ε2 alleles with the studied biomarker profile. The E2/E2 genotype was excluded from statistical analysis primarily due to the limited number of participants (*N* = 1) exhibiting this genotype in our study population. For the E2/E3 genotype, a weak negative correlation among triglycerides, HDL, APOA, SDLDL, APOB:APOA, PLAC, and Apo-B was observed. Conversely, there was a weak positive correlation among LDLCAL, MPO, OXLDL, BNPNT, HOMOC, hs-CRP, LPA, LDL, and cholesterol. E2/E4 carriers exhibited a weak positive correlation with BNPNT, SDLDL, OXLDL, LDLCAL, and PLAC and exhibited a moderate positive correlation with hs-CRP and MPO. The lipid parameters LDL, HDL, APOB, LPA, and HOMOC showed weak negative correlations with E2/E4 carriers, while triglycerides, APOB:APOA, and APOA showed a weak positive correlation. 

**Table 3:** Pearson correlation of APOE genotypes with vital lipid and inflammatory biomarkers. Pearson correlation coefficients were calculated to assess the relationship between APOE genotypes and various lipid and inflammatory biomarkers. The data are presented as Pearson correlation coefficients (*r*), with significance levels set at *P* < 0.05, indicating significant correlations between APOE genotypes and biomarker levels. LDL, low-density lipoprotein; HDL, high-density lipoprotein; APOA, apolipoprotein A; APOB, apolipoprotein B; Lp(a), lipoprotein A; hs-CRP, high-sensitivity C-reactive protein; HOMOC, homocysteine; BNPNT, B-type natriuretic peptide; SDLDL, small-density LDL; OXLDL, oxidized low-density lipoprotein; MPO, myeloperoxidase; LDLCAL, LDL calculated; PLAC, lipoprotein-associated phospholipase (Lp-PLA2) test

	E2/E3		E2/E4		E3/E4		E4/E4	
Parameters (mmol/L)	r	P	r	P	r	P	r	P
Cholesterol	0.104	0.576	-0.134	1.000	-0.030	0.455	-0.045	0.1943
LDL	0.032	0.194	-0.176	0.413	-0.038	0.381	-0.175	0.0268
HDL	-0.048	0.723	-0.335	0.922	-0.072	0.893	0.095	0.9027
Triglycerides	-0.236	0.557	0.377	0.250	-0.068	0.666	-0.193	0.9314
APOA	-0.024	0.575	-0.457	0.931	-0.109	0.987	0.266	0.4948
APOB	-0.050	0.000	-0.187	0.323	-0.052	0.448	-0.233	0.1361
Lp(a)	0.052	0.907	-0.046	0.955	-0.060	0.863	0.030	0.8534
hs-CRP	0.075	0.708	0.429	0.537	-0.047	0.920	0.132	0.8118
HOMOC	0.193	0.973	-0.219	0.403	0.037	0.772	-0.476	0.8244
BNPNT	0.053	0.894	0.118	0.594	-0.022	0.950	-0.224	0.7847
SDLDL	-0.184	0.460	0.268	0.788	-0.045	0.692	-0.006	0.3527
OXLDL	0.012	0.877	0.038	0.613	-0.063	0.905	0.247	0.4780
MPO	0.056	0.966	0.562	0.556	-0.107	0.959	0.550	0.8270
APOB:APOA	-0.260	0.184	-0.357	0.997	-0.070	0.527	0.146	0.1062
LDLCAL	0.015	0.316	0.111	0.568	-0.044	0.354	0.103	0.1126
PLAC	-0.079	0.469	0.133	0.629	0.050	0.411	-0.060	0.0871

The assessment of the ε3 genotypes revealed that E3/E4 carriers had a weak negative correlation with total cholesterol, LDL, HDL, triglycerides, Lp(a), hs-CRP, BNPNT, SDLDL, ox-LDL, MPO, APOB:APOA, LDL-CAL, APOA, and APOB. The analysis also showed a weak positive correlation with HOMOC and PLAC. 

On assessing the ε4 genotypes, E4/E4 carriers were found to have a moderate negative correlation with HOMOC and a weak negative correlation with markers including, total cholesterol, LDL, triglycerides, BNPNT, SDLDL, PLAC, and APOB. Conversely, the genotype observed a weak positive correlation with HDL, APOA, Lp(a), hs-CRP, OXLDL, APOB:APOA, and LDL-CAL levels. Additionally, E4/E4 carriers exhibited an increase in inflammatory levels with a moderate positive correlation with MPO levels. 

Based on correlation analysis, we observed significant associations in LDL levels in the E4/E4 genotype and APOB levels in the E2/E3 genotype. Specifically, we found that LDL levels in E4/E4 carriers showed significant differences than the E3/E3 genotype (*P *= 0.02). Similarly, APOB levels in E2/E3 carriers was significantly different compared to the E3/E3 genotype (*P *= 0.00). However, the analysis did not reveal significant associations for the other lipid and inflammatory markers studied.

## Discussion

APOE, produced in the CNS and the periphery, is a multifunctional protein-regulating lipid metabolism, specifically, the transport and redistribution of cholesterol and other lipids throughout the body [21]. Of the three major APOE alleles, the ε4 allele is particularly associated with altered lipid profiles, increased LDL levels, decreased HDL levels, and disrupted cholesterol metabolism. These abnormalities contribute to the development of atherosclerosis and the accumulation of cholesterol in the brain, in the case of AD [22]. The ε4 isoform also influences inflammatory markers, such as CRP, leading to heightened inflammation [23]. The interplay between APOE gene polymorphisms, lipid abnormalities, and inflammation is associated with the risk of CVD and AD [24]. 

In the present study, the APOE genetic profile was assessed using the biomarker levels for the E3/E3 genotype as a reference due to its dominant prevalence in the general population. The results revealed that individuals with the E2/E3 genotype exhibited high levels of triglycerides, along with high levels of the protective markers, HDL and APOA. This genotype was also associated with elevated levels of inflammatory markers including hs-CRP and MPO. The E2/E3 genotype demonstrated negative associations with most lipid and inflammatory markers. Additionally, the E2/E4 genotype was associated with high levels of HDL, triglycerides, Lp(a), and BNPNT, whereas they were associated with low levels of cholesterol, LDL, APOA, APOB, SDLDL, OXLDL, APOB:APOA, LDLCAL, and all the inflammatory markers. Previous studies have indicated that E2/E3 increased the levels of *good* cholesterol indicators like HDL cholesterol and APOA, thereby potentially reducing the risk of AD and CVD [25]. In addition, favorably low levels of LDL were observed in the E2/E3 and E2/E4 genotypes. Nevertheless, these genotypes also witnessed an increase in the risk-associated triglyceride levels. Similar findings were observed in previous studies; however, the mechanism driving the change in the lipid levels is still to be elucidated [25]. 

This study revealed that the E3/E4 genotype is associated with elevated levels of several lipid and inflammatory markers, including cholesterol, LDL, HDL, APOB, Lp(a), SDLDL, OXLDL, APOB:APOA, LDLCAL, hs-CRP, HOMOC, MPO, and PLAC and lower levels of triglycerides, APOA, and BNPNT. Although there was a marginal increase in HDL levels in E3/E4 carriers (60.56 ± 21.028 mg/dL) compared to the reference genotype E3/E3 (59.8 ± 17.47 mg/dL), this increase may not confer significant protective effects. Overall, these observations suggest that individuals carrying the E3/E4 genotype are prone to higher levels of risk markers linked to CVD and AD. For the E4/E4 genotype, elevated levels of cholesterol, LDL, APOB, Lp(a), SDLDL, OXLDL, APOB:APOA, and LDLCAL, hs-CRP, HOMOC, MPO, and PLAC were observed. Nevertheless, this genotype also had low levels of HDL, APOA, triglycerides, and BNPNT markers. These findings are consistent with previous research [26] indicating that individuals with the ε4 allele tend to have higher concentrations of LDL and cholesterol, thereby increasing the risk of developing CVDs or AD. The regulation of cholesterol transport and localization within the body mediated by APOE plays a crucial role in CVD and AD as it is a component of atherosclerotic and amyloid-beta plaques [26]. Moreover, since a significant portion of APOB and Lp(a) in plasma is typically bound to LDL, it is not surprising that individuals carrying the ε4 allele exhibited the highest levels of APOB [27,28]. Previous studies have suggested alterations in APOB, Lp(a), and LDL levels in both AD and CVD. Further investigation is needed to understand the involvement of APOB and Lp(a) in cellular pathways associated with disease pathogenesis [29,30].

To our knowledge, the present study is the first to discuss the levels of APOB:APOA, LDL-CAL, and PLAC in the serum of different APOE genotypes. It was found that the ɛ4 allele-carrying individuals typically had higher levels of APOB:APOA, LDL-CAL, and PLAC in their serum, with the descending order, E4/E4 > E3/E4 > E3/E3 > E2/E4 > E2/E3. Previous studies suggest that the elevated APOB:APOA is indicative of impaired LDL clearance and a higher abundance of LDL particles contributing to cholesterol accumulation. Higher LDL-CAL levels reflect impaired LDL clearance, while increased PLAC levels indicate a propensity for plaque development [31]. It is important to acknowledge that the presence of these risk markers in the E3/E4 and E2/E4 genotypes could be attributed to the autosomal dominance of the ε4 allele. This implies that a single ε4 allele can significantly alter lipid levels, leading to an increased risk of CVD and AD [32]. 

Using Pearson correlation analysis, the comparison of each genotype with E3/E3 revealed significant biomarker correlations for the E2/E3 and E4/E4 genotypes. The E2/E3 genotype had significantly lower levels of APOB (*r* =-0.05, *P *= 0.00), while it showed a nonsignificant correlation with LDL levels. On the other hand, the E4/E4 genotype had significantly higher levels of LDL (*r *= -0.175, *P *= 0.02), while it had a nonsignificant association with APOB. The significant association of the E2/E3 genotype with low levels of the risk factor, APOB, and the E4/E4 genotype with high levels of the risk factor, LDL, highlight the differential effects exerted by APOE alleles on lipid levels in the body. Previous research has indicated that the ε2 allele is associated with lower APOB levels and the ε4 allele is associated with higher APOB levels when compared to the ε3 allele, respectively [33]. Additionally, it is well-established that ε4 allele carriers have higher LDL and lower HDL levels compared to non-carriers [34]. Although not significant, our study observed a weak positive correlation in the ε4 group for low levels of HDL. The findings from this study help in fortifying the observed change in lipoprotein levels brought about by the APOE alleles. Elucidating the mechanisms through which these alleles confer risk and influence biomarker levels can facilitate the establishment of genotype-specific biomarker profiles that may help catalyze advancements in therapeutic strategies. 

This study effectively highlights that the APOE4 genotype confers an increased risk for CVD and AD complications through dysregulated lipid metabolism and abnormal inflammatory profiles. Genetic APOE testing can be used as an early screening tool for CVD and AD risk assessment. The feasibility of genetic testing in early risk assessment makes it an attractive prospect for CVD and AD screening as it can be done from the comfort of one’s home. Saliva or Dried Blood Spot kits are available for easy sample collection and extraction of DNA for genetic assessment by diagnostic laboratories [35]. The convenience related to the accessibility and feasibility of APOE testing via test kits might help individuals get familiarized with the test kits as early tools for disease risk assessment. This may prospectively lead to increased early CVD and AD risk screening which may enable early diagnosis and the implementation of personalized interventions, including targeted dietary and lifestyle modifications [36]. For APOE4 carriers, a rigorous intervention involving dietary, supplement, and lifestyle practices can be implemented to manage lipid abnormalities and inflammation [37]. This may include following a Mediterranean diet, incorporating omega-3 fatty acids, increasing fiber intake, avoiding smoking, and exercising regularly [38]. Conversely, APOE2 carriers, who are at lower risk, can focus on consuming a balanced diet and adopting healthy lifestyle practices to optimize their health. 

The study has several limitations that must be acknowledged. One limitation is the unequal sample sizes among the genotypes, which may have hindered our ability to observe robust correlations and could have contributed to the lack of statistical significance by reducing our statistical power. We also recognize that our results may have been influenced by other unmeasured or uncontrolled factors like lifestyle choices (e.g., diet, exercise) and environmental influences (e.g., stress levels). Furthermore, genetic variations beyond the APOE genotype which were not considered in our study could have influenced the observed weak correlations or the non-significant results thereof. The absence of ethnic diversity in our study is another limitation that could have affected the effectiveness of the results. Additionally, as our study was a one-time assessment, it lacks follow-up data that could provide further support for the associations identified. To strengthen the results obtained from this study, follow-up analyses with larger samples must be conducted. 

## Conclusions

In conclusion, the present study provides compelling evidence linking APOE gene polymorphisms to abnormal serum lipid and inflammatory profiles. Mutations in the gene change the three-dimensional structure of the APOE protein and its lipid-carrying capability. Specifically, individuals carrying the ε4 alleles exhibited dysregulated lipid metabolism and abnormal inflammatory markers profile which tended towards increased CVD and AD risk, while ε2 alleles tended toward decreased risk. These findings underline the importance of early detection, prompt diagnosis, and standardized therapeutic approaches to mitigate the risk of diseases related to abnormal lipid and inflammatory profiles. APOE testing via simple at-home collection using saliva or dried blood spot samples is a convenient way of assessing risk for CVD and AD. By prioritizing proactive measures in at-risk individuals, we can effectively reduce the incidence and severity of lipid and inflammation-related disorders.
